# Validation of the effectiveness of pig farm repopulation protocol following African swine fever outbreaks in the Philippines

**DOI:** 10.3389/fvets.2024.1468906

**Published:** 2024-12-09

**Authors:** Chia-Hui Hsu, Maximino Montenegro, Ruth Miclat-Sonaco, Montserrat Torremorell, Andres M. Perez

**Affiliations:** ^1^Center for Animal Health and Food Safety, College of Veterinary Medicine, University of Minnesota, Saint Paul, MN, United States; ^2^Department of Veterinary Population Medicine, University of Minnesota, Saint Paul, MN, United States; ^3^Pig Improvement Company (PIC) Philippines, Pasig City, Philippines; ^4^National Livestock Program, Office of the Undersecretary for Livestock, Department of Agriculture, Quezon City, Philippines

**Keywords:** African swine fever, survival analysis, early detection, repopulation, epidemiology, sensitivity, Philippines, policy

## Abstract

The African swine fever (ASF) epidemic has severely challenged the Philippines’ swine industry since 2019. The National African Swine Fever Prevention and Control Program (NASFPCP), launched in 2021, aims to provide guidance for managing ASF through surveillance, monitoring, and swine repopulation. This study evaluates the effectiveness of post-outbreak disinfection protocols and government-mandated measures for repopulation standard. Surveillance data from three repopulation phases—(I) depopulation, cleaning, and disinfection; (II) downtime (20 days); and (III) sentinel animals (40 days)—were collected from February 2020 to December 2021 in the province of Batangas. Time-to-detection of positive events were analyzed for different farm types, seasons, or location using survival analysis modeling. Probability of detecting infected farms at different sampling times was estimated in terms of sensitivity of the sampling time. Data from 145 swine farms, including 99 backyard and 46 commercial farms, revealed positive rates of 10.1 and 8.7%, respectively. The failure rate during repopulation surveillance was 9.66%, whereas 90.34% farms remained ASF negative. Sensitivity estimate increased from 18–21 to 89% by day 27, with sentinel animals on that day exhibiting the highest estimated sensitivity. This highlights the importance of sentinel pigs in the NASFPCP for effective ASF control in the Philippines. Survival analysis showed no statistically significant differences in the results between either farm type, season, or municipality level. Geographic mapping of surveyed farms and those with positive detections identified high-risk locations including San Juan and Lipa City as key areas of concern. Enhancing targeted surveillance is critical for improving an early ASF detection and national response in the Philippines.

## 1 Introduction

African swine fever (ASF), caused by the African swine fever virus (ASFV), is a severe transboundary animal disease first described in Kenya by Montgomery (1). ASFV continues to spread globally, impacting pig health and welfare. It has affected several countries in Asia, the Caribbean, Europe, and the Pacific, impacting both domestic and wild pigs. This acute, hemorrhagic and highly contagious DNA virus targets swine populations, often resulting in mortality rates nearing 100%. While ASF does not pose a direct threat to human health, its impact on swine herds is economically devastating, influencing rural development and food security, and leading to considerable socio-economic challenges in affected countries (2–5). As of 2024, the disease has affected 19 countries across the Asia-Pacific region (6), primarily dominated by ASFV genotype II.

ASF spread rapidly across Southeast Asian countries between 2019 and 2023. In the Philippines, ASF was first reported in July 2019 in Rizal province near Manila (7). Since then, the disease has escalated with varying levels of severity, evolving from a localized outbreak to regional and national levels, and it continues to persist as of July 2024. Several infected areas have maintained or expanded their red zone status—areas with confirmed ASF cases—leading to substantial losses in the swine industry (8). Prior to the ASF outbreaks, the Philippine Statistics Authority reported 12.70 million pigs in 2019 (9). However, by September 2023, this number had dropped to 9.86 million, a 23% decrease (10). This shortage also led to a significant increase in the farmgate price of pork, rising from PhP 110.52 (US$1.92) per kg in early 2019 to PhP 170.26 (US$2.95) per kg in early 2023, a 54% increase (11). These statistics underscores the severe impact of ASF on the country’s swine industry.

Currently, the focus of managing ASF control is shifting from managing ASF as an epidemic to addressing it as an endemic disease, aiming to minimize its impact and enhance repopulation efforts. Since the inception of the National ASF Prevention and Control Program (NASFPCP, formerly BABay ASF), guidelines have been in place for implementing ASF surveillance, monitoring, and swine repopulation procedures, extending to the local government level. This effort also includes standardized protocols for swine repopulation (12, 13). Administrative Order No. 6 and No. 7, Series of 2021, issued by the Office of the Secretary, details the repopulation protocols in response to ASF outbreaks in the Philippines, highlighting a critical area of interest for both the government and researchers.

**Figure 1:**
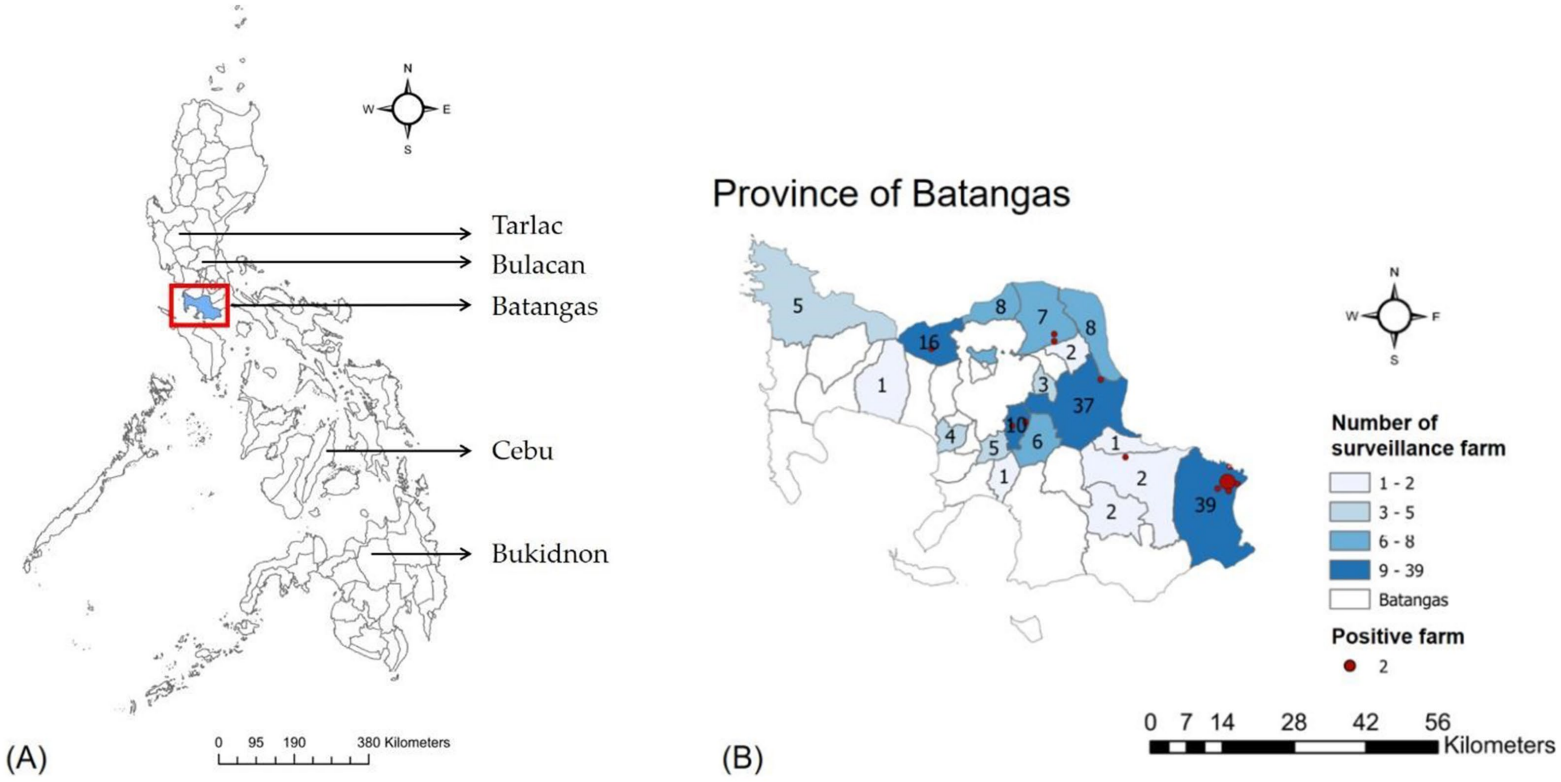
**(A)** The map of the Philippines with Batangas highlighted by a red rectangle. **(B)** The number of surveillance farms in each city/municipality, with positive farms represented as dots.

In the Philippines, Batangas province stands out as the primary provincial source of hogs, contributing 8% of the nation’s total swine output (14). Other provinces of significant production include Bulacan (7%), Bukidnon (6%), Cebu (5%), and Tarlac (4%) (Figure 1A). The first ASF case in Batangas province was recorded in Laurel Municipality in February 2020. According to the 2018 Swine Situation Report by the Philippine Statistics Authority, Batangas ranked as the second-largest hog-producing region in the country, with an output exceeding 174,000 metric tons. The ASF outbreak in Batangas has had a profound impact on the nation, emphasizing the crucial need for meticulous documentation of farm-level records. Such documentation aids in investigating follow-up repopulation efforts, serving as an example to assess the efficacy of government-mandated measures and the impact of disinfection and cleaning protocols post-ASF outbreak.

This study aims to assess the effectiveness of national repopulation protocols by analyzing surveillance outcomes of sentinel animals and estimating the sensitivity and specificity of ASF tests at the farm level. It also aims to leverage data-driven insights to enhance recovery efforts following ASF outbreaks in Batangas, Philippines. The overarching goal is to enhance targeted surveillance and early detection of ASF, thereby bolstering the country’s response to ongoing ASF outbreaks.

## 2 Materials and methods

### 2.1 Data

**Figure 2:**
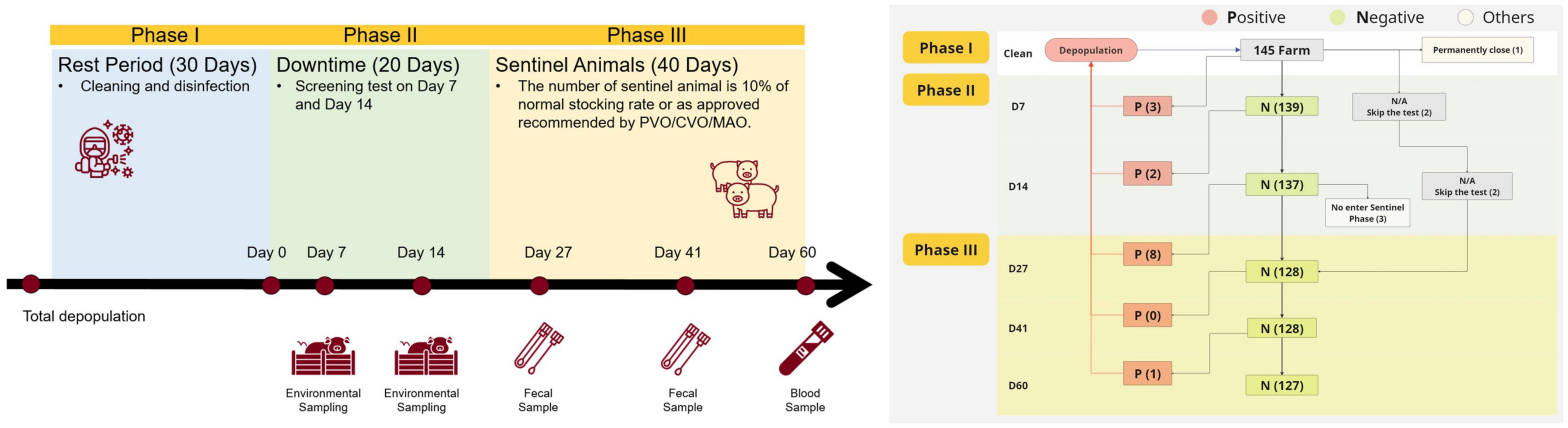
**(A)** The framework for recovery and repopulation is established according to Administrative Order No. 06, Series of 2021. It outlines the repopulation, biosecurity, monitoring, and surveillance protocols as per the Department of Agriculture, Philippines. **(B)** The flowchart illustrates 5 rounds of ASF testing on different samples (including environmental, fecal, and blood) and the response protocol for swine farms following an ASF positive detection event during different phases. The flowchart outlines the step-by-step process from initial depopulation after an ASF outbreak through to the end of day 60 surveillance (phase III), emphasizing the stringent testing measures at multiple stages. The protocol begins with the depopulation of all pigs on ASF-affected farms. At each testing phase, farms are either identified as negative and allowed to proceed to the next stage, or positive, triggering immediate depopulation. Farms consistently testing negative through all 5 time points are eventually deemed safe for future repopulation.

Farm-level data for the study were collected from February 13, 2020 (the first detection of ASF in Batangas), to December 21, 2021, and provided by the Provincial Office of Batangas City, Philippines. Data were collected, including farm demographics such as whether the farm was categorized as backyard or commercial, farm unique ID, farmer’s name, each administrative level of the farm (region, province, municipality/city, barangay), date of the first ASF outbreak at farm, diagnostic tests required at each surveillance time point, and the corresponding results indicating positive or negative findings. Additionally, any follow-up comments by government officers were recorded. The framework for surveillance, recovery, and repopulation is outlined in Figure 2.

### 2.2 Repopulation protocol

Each surveillance farm participating in this study followed a mandatory three-phase government repopulation protocol lasting a cumulative of 90 days. The protocol included guidance, technical support, and supervision by government veterinary officers (15).

### 2.3 Phase I: depopulation, cleaning and disinfection (rest period, 30 days)

In response to an ASF outbreak on a farm, all domestic pigs on the affected farm are culled, initiating a 30-day rest period. During this time, organic debris is removed, movable or non-movable equipment is cleaned, and lagoons and manure pits are emptied. Bird netting and rat control are implemented. Disinfection involves using foaming agents to improve the effectiveness of disinfectants, focusing on areas likely to harbor the virus, such as feeders, drinkers, and dunging areas. Additional treatment, using only approved disinfectants for ASF control, includes caustic soda and hydrated lime, ensuring proper dilution, coverage, and contact time (15).

### 2.4 Phase II: downtime (20 days)

After cleaning and disinfection, a further 20-day downtime period reduces bacterial load and residual ASF virus risk. Environmental sampling is conducted, targeting well water, topsoil at burial sites, and various surfaces across the farm (gestating pens, farrowing pens, hallways and stock rooms). Initial screening for ASF is performed on day 7 using loop-mediated isothermal amplification (LAMP) with samples collected from a 500-meter radius around the infected farm. Samples are collected and submitted to the Regional Animal Disease Diagnostic Laboratory (RADDL) by the assigned Provincial, City, or Municipal Veterinary Officer. A second screening occurs on day 14, with PCR validation for positive results. If both screenings are negative, the farm is eligible to proceed to phase III, allowing for the introduction of sentinel animals.

### 2.5 Phase III: introduction of sentinel animals (40 days)

Sentinel animals (60-day-old piglets weighing 15–20 kg) are sourced from ASF-free farms and introduced to the cleaned farm. The number of sentinel pigs is set at 10% of the original stocking inventory. Fecal swab samples from all restocked pigs are collected on day 27 and day 41 for ASF testing, validated with PCR if positive. If confirmed, depopulation is triggered. A final test is performed on day 60 with blood samples for PCR testing. Sentinel animals follow the all-in, all-out principle for disposal. If results are negative, the repopulation process continues as outlined.

### 2.6 Case definition

During the 60-day monitoring period (phases II: day 0–day 20 and III: day 21–day 60, shown in Figure 2A), consistent ASF-negative results were used to indicate effective cleaning and disinfection, negative farms (NF). If any point test returned a positive result, it was confirmed by PCR and recorded as an ASF-positive detection event and the farm was denoted as positive (positive farm, PF). We then estimated the proportion of farms where the protocol failed (defined as failure rate), indicated by the presence of ASFV through sampling, among the total number of assessed farms (TF), as ∑PF/TF. This involved documenting ASFV presence and recording time-to-detection data, focusing on the interval from the completion of cleaning and disinfection (end of phase I) to the first ASF-positive detection.

### 2.7 Calculation of sensitivity and specificity

Sensitivity (Se) and specificity (Sp) were calculated at each sampling point *i* as PF*i*/(∑PF) and NF*i*/(∑NF), respectively. For this study, it was assumed that there was no external introduction of ASF and that new cases were due to insufficient contamination control. It was also assumed that no false negatives occurred after day 60 (D60), the day the blood sample was tested using PCR. The results of Se and Sp varied across different testing days.

### 2.8 Survival analysis modeling

To evaluate whether results were affected by different factors such as farm type, seasonality, or location, we conducted a survival analysis using the R statistical software (package survival). We generated Kaplan–Meier survival curves to graphically represent the progression of ASF-positive cases over time across different categorical variables. These curves visually depict the timing and frequency of ASF detections throughout the observation period.

## 3 Results

During the study period, a total of 145 swine farms were included in the analysis. One farm permanently closed, whereas 14 farms experienced positive detections (from environmental screening and fecal samples) during the surveillance period and immediately underwent disinfection, with total depopulation if they were in phase III. These positive farms re-entered the surveillance process from the beginning, resulting in a duration exceeding 60 days of observation. Despite this rigorous process, all these farms maintained negative results upon retesting until the end of the surveillance period. Thus, a total of 158 (145 – 1 + 14) farm-level data points were recorded. Overall, 90.34% (131/145) of the farm-level surveillance showed negative detection of the ASF virus.

Of the total 145 farms, 99 were backyard farms and 46 were commercial farms. Among the backyard farms, 10.1% (10/99) tested positive in at least one of the five diagnostic tests, while 8.7% (4/46) of the commercial farms tested positive. During the sentinel animal phase, following the guideline of selecting 10% of the normal stocking rate, the median number of sentinel pigs across the 145 farms was 3, with a maximum of 25 and a minimum of 1.

Sensitivity (Se) and Specificity (Sp) value represents a point estimate, as shown in Table 1. For example, on D7, sensitivity was 21%, calculated as 3 true positives out of a total of 14 (3 true positives plus 2 + 8 + 1 false negatives). On D14, sensitivity was 18%, with 2 true positives out of 11 (2 true positives plus 8 + 1 false negatives). Sensitivity significantly increased to 89% by D27, reflecting 8 true positives out of 9 (8 true positives plus 1 false negative), indicating improved detection as the protocol progressed. Additionally, cumulative sensitivity was also calculated to provide a broader understanding of the protocol’s effectiveness over time.

**Table 1:** This table summarizes ASF testing results across each surveillance periods (day 7, day 14, day 27, day 41, and day 60) in backyard and commercial swine farms.

	Day 7	Day 14	Day 27	Day 41	Day 60
Backyard positive (a)	1	1	7	0	1
Commercial positive (b)	2	1	1	0	0
Subtotal positive (c, a + b)	3	2	8	0	1
Negative (d)	139	137	128	128	127
Total detection (c + d)	142	139	136	128	128
Prevalence (c/c + d; positive %)	2.11%	1.43%	5.88%	0	0.78%
Sensitivity (Se)	21%	18%	89%	0	100%
Cumulative sensitivity (cum Se)	21%	36%	93%	93%	100%
Specificity (Sp)	92%	93%	99%	99%	N/A

It includes counts of positive detections, prevalence, sensitivity, cumulative sensitivity, and specificity point estimates for each farm type.

The province of Batangas has 34 cities or municipalities, of which 17 had surveillance farms included in the analysis. Figure 2 displays the geographic distribution of surveillance farms in each city or municipality, along with the number of positive farms (represented as proportional dots). San Juan, located in the southeast on the map, has the highest number of surveillance farms, with 39 farms, followed by Lipa City with 37 farms. San Juan also has the most positive farms, with a total of 7.

### 3.1 Survival analysis modeling for categories

**Figure 3:**
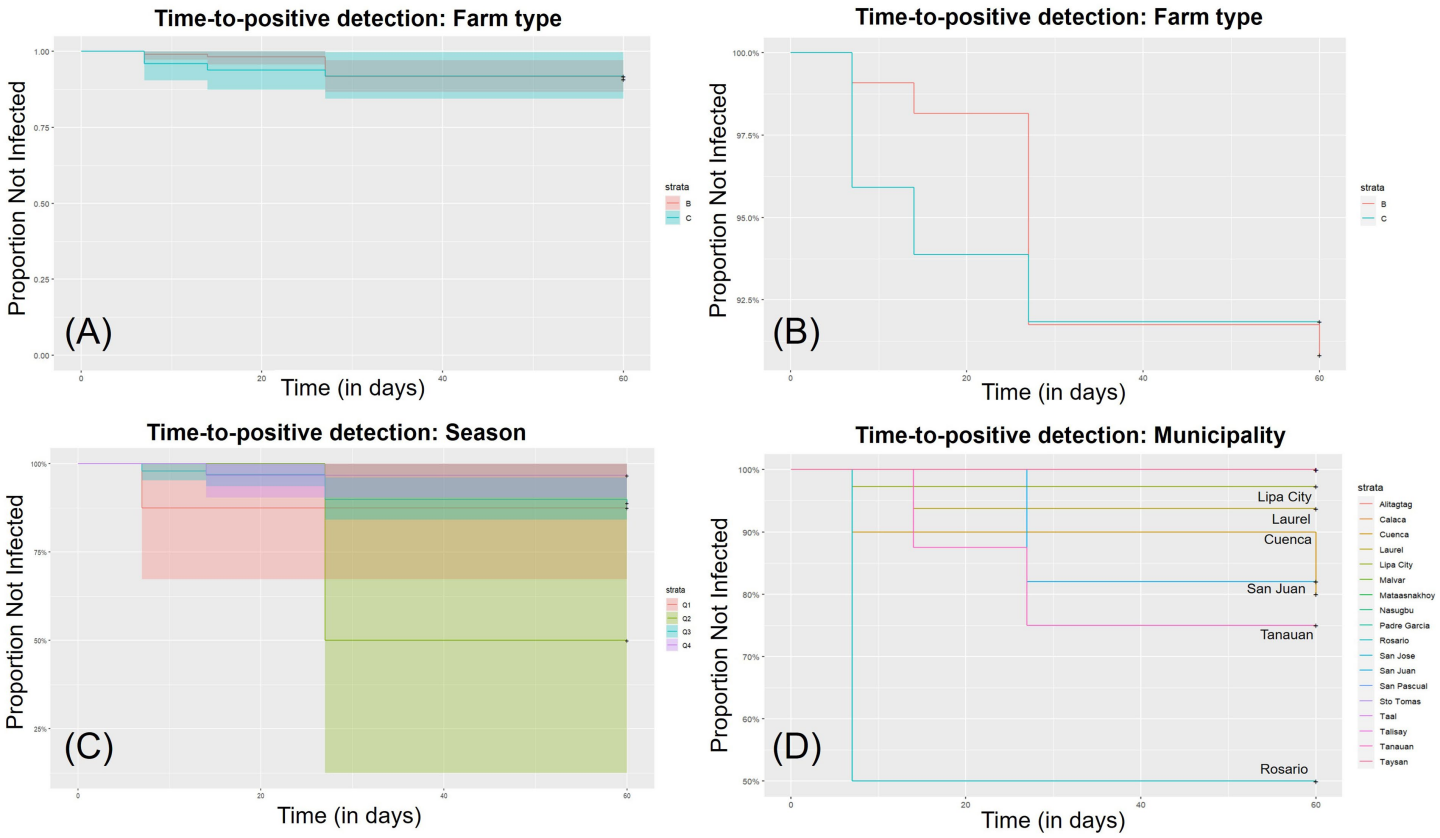
Survival analysis of field samples across various categorical variables. **(A,B)** Show comparison between backyard and commercial farms, both with over 90% negative detection rates and overlapping 95% confidence intervals, indicating no significant difference. **(C)** Compares seasonal quarters: Q1 (January–March), Q2 (April–June), Q3 (July–September), and Q4 (October–December). **(D)** Compares different municipalities/cities, each represented by different line colors. Overlapping 95% confidence intervals in all panels suggest no significant differences across the variables.

A survival analysis was conducted to illustrate the time to positive detection events, indicating when ASF was detected on the farm (Figure 3). The results suggest that there is no significant difference between backyard and commercial farms. At the municipality or city level, the analysis shows the timing of positive ASFV detections during the three surveillance phases (Figure 3D), with no significant difference between different quarters and municipalities.

## 4 Discussion

The National ASF Prevention and Control Program repopulation protocol implemented in Batangas indicates that 90.34% of farm-level surveillance reported no detection of the ASF virus over the post-outbreak surveillance period. However, there is an approximate 10% failure rate among farms, suggesting some challenges in protocol adherence or effectiveness. Ideally, thorough appropriate cleaning, disinfection, and strict biosecurity measures should ensure virus prevention across all farms. Our findings highlight the need for consistent and rigorous compliance with comprehensive biosecurity protocols across different municipalities and cities to effectively mitigate virus persistence despite disinfection efforts.

Based on sensitivity estimates on day 27 (with 89% point estimate and 93% cumulative sensitivity), in this study, fecal samples show better performance compared to environmental samples taken on day 7 and day 14 under field conditions. The effectiveness of sentinel animals in detecting ASFV during phase III underscores the importance in the repopulation protocol, surpassing the efficacy of environmental sampling alone. This research provides fundamental information for the central government in determining appropriate comparative evaluation and sampling methods for the National ASF Prevention and Control Program. While the national guidelines suggest assumption of 6% prevalence rate for an ASF-infected red zone (16), this may currently be underestimated given the unknown exact prevalence. Therefore, even if every province strictly follows the protocol, the sampling method and number should be estimated based on the local prevalence rate.

Batangas province serves as a representative example of the broader Philippine scenario. The ratio of backyard farms to commercial farms in our research is 68.3% (99/145) versus 31.7% (46/145), which closely resembles the national ratio. Recent retrospective research from the Philippines indicates that smallholder farms are about 3.85 times more likely to be infected with ASF compared to commercial farms (12). However, our analysis in Batangas showed no statistically significant difference between farm types. This finding indicate that the current repopulation protocol may be effective for both farm types.

Previous research suggests that ASF outbreaks in the Philippines exhibit seasonal patterns and are related to spatial factors, with different risk levels associated with backyard and commercial farming (12, 17). To validate these findings, we included these categorical variables in our survival analysis model. While we did not find significant results for all variables, we observed a notable aggregation of detections in San Juan municipality, southeast Batangas, where multiple farm outbreaks occurred (Figure 1B). This highlights a potential hotspot that warrants further investigation. Some positive farms may be involved in distributive farming, which could lead to downstream infection spread and a domino effect of infections. Therefore, strict control measures must be implemented at these farms. While conclusive evidence remains elusive, local government epidemiological investigations suggest that ASF infections may be linked to hog traders operating across provinces. The outbreak in San Juan possibly originated from Quezon Province, where ASF outbreaks were confirmed earlier. Animal haulers frequently purchased and transported hogs from or through Quezon Province to San Juan, likely contributing to the continuous spread of the infection. In addition, seasonal flooding in the Philippines, including the Malaking Tubig River across San Juan Municipality, may also potentially facilitate local ASF transmission. A critical issue in the Philippines is the lack of traceable hog data due to highly reliance on paper-based documentation in local government units, hindering real-time access and overall coordination. The Bureau of Animal Industry has a Philippine Animal Health Information System (Phil-AHIS) unit (18), which is currently underutilized. Strengthening this unit and implementing a centralized digital system is crucial for improving infection tracing and response effectiveness.

In the Philippines, the registration of backyard farmers began at the local government unit only after the ASF outbreak in 2019, with incentives such as free feed, livestock management education, and swine disease vaccinations to encourage participation. Despite these efforts, the exact number of registrations remains uncertain, particularly in remote mountainous regions where program awareness is low, and misconceptions about ASF persist. This indicates an inherent bias in our sampling surveillance protocol, as resource availability varies significantly across provinces. Batangas stands out as a vital province in region IV-A, benefiting from ample resources, proximity to Metro Manila. It was also the first province declared free of ASF after a 16-month battle (19), although it experienced a re-outbreak later. The capacity of veterinary officers and compliance with surveillance protocols make Batangas ideal for assessing the effectiveness of national biosecurity measures. However, caution is needed when applying these findings to resource-limited regions. Besides, Batangas is geographically connected to three other provinces in the region and serves as the swine hub. Evaluating the parallel data from these connected provinces will provide a holistic view of transportation and enhance the understanding of ASF spread through various human behaviors.

A significant assumption in this research is that ASFV detection was attributed to potential failures in the cleaning and disinfection rest period during phase I of the repopulation protocols. However, uncertainty remains regarding whether these detections could also result from new introductions during personnel sampling or other environmental factors. Despite this assumption, stringent measures were implemented during sampling, including the use of proper personal protective equipment (PPE) and adherence to specific biosecurity protocols. Trained personnel, such as Barangay Biosecurity Officers (BBO), assisted in the sampling process. Additionally, all sentinel animals were sourced from ASF-free farms and monitored before introduction to the farms under repopulation.

In conclusion, our study in Batangas Province highlights the effectiveness of current repopulation protocols in controlling ASF, with 90.34% of farms achieving negative ASF detection. Sentinel animals were more effective than environmental sampling. Tailored sampling strategies based on local adoption, needs, and resources are crucial. Implementing a centralized digital data system is essential for enhancing nationwide outbreak response. Addressing human factors and improving biosecurity compliance are vital for managing and controlling ASF in the Philippines.

## Data Availability

Data are the property of the Philippines government and have been shared with us to conduct the research here to support training activities in the country. Any requests should be directed to the Philippine Bureau of Animal Industry and to the International Training Center on Pig Husbandry of the Philippines.
